# Cultivar-specific transcriptome and pan-transcriptome reconstruction of tetraploid potato

**DOI:** 10.1038/s41597-020-00581-4

**Published:** 2020-07-24

**Authors:** Marko Petek, Maja Zagorščak, Živa Ramšak, Sheri Sanders, Špela Tomaž, Elizabeth Tseng, Mohamed Zouine, Anna Coll, Kristina Gruden

**Affiliations:** 1 Department of Biotechnology and Systems Biology, National Institute of Biology, Ljubljana, Slovenia; 2 Jožef Stefan International Postgraduate School, Ljubljana, Slovenia; 3 National Center for Genome Analysis and Support (NCGAS), Indiana University, Bloomington, USA; 4 PacBio, Menlo Park, CA USA; 5 Laboratoire Génomique et Biotechnologie des Fruits, INRA-INP/ENSAT, Castanet-Tolosan, France

**Keywords:** Genome informatics, Standardization, Plant sciences, Data processing, Transcriptomics

## Abstract

Although the reference genome of *Solanum tuberosum* Group Phureja double-monoploid (DM) clone is available, knowledge on the genetic diversity of the highly heterozygous tetraploid Group Tuberosum, representing most cultivated varieties, remains largely unexplored. This lack of knowledge hinders further progress in potato research. In conducted investigation, we first merged and manually curated the two existing partially-overlapping DM genome-based gene models, creating a union of genes in Phureja scaffold. Next, we compiled available and newly generated RNA-Seq datasets (cca. 1.5 billion reads) for three tetraploid potato genotypes (cultivar Désirée, cultivar Rywal, and breeding clone PW363) with diverse breeding pedigrees. Short-read transcriptomes were assembled using several *de novo* assemblers under different settings to test for optimal outcome. For cultivar Rywal, PacBio Iso-Seq full-length transcriptome sequencing was also performed. EvidentialGene redundancy-reducing pipeline complemented with in-house developed scripts was employed to produce accurate and complete cultivar-specific transcriptomes, as well as to attain the pan-transcriptome. The generated transcriptomes and pan-transcriptome represent a valuable resource for potato gene variability exploration, high-throughput omics analyses, and breeding programmes.

## Abstract (summary)

**Table:** 

Measurement(s)	genome • RNA • sequence_assembly • transcriptome
Technology Type(s)	digital curation • RNA sequencing • sequence assembly process
Factor Type(s)	cultivar
Sample Characteristic - Organism	Solanum tuberosum

Machine-accessible metadata file describing the reported data: 10.6084/m9.figshare.12562733

## Background & Summary

At species level, genomes of individuals can differ in single nucleotide polymorphisms (SNPs), short insertions and deletions (INDELs), gene copy numbers, and presence or absence of genes^1^. The latter leads to the concept of species specific pan-genomes, namely the core genome present in most individuals and the dispensable genome comprised of genes present only in a subset of individuals, which results in the emergence of particular subgroup-specific phenotypes. This concept has been extended to pan-transcriptomes, where the presence or absence of variations is not bound only to gene content, but also to genetic and epigenetic regulatory elements. Pan-genomes and pan-transcriptomes have been described in the model plant species *Arabidopsis thaliana*^2^ and several crop species, including maize^3,4^, rice^5^, wheat^6^ and soybean^7^.

While the genome of a double-monoploid clone of *Solanum tuberosum* Group Phureja (DM) is available^8^, this diploid potato group differs from the tetraploid Group Tuberosum, which includes most varieties of cultivated potato. Through domestication and modern breeding efforts, different potato cultivars also acquired genes from other closely related Solanum species or lost some ancestral genes^1^. Different breeding programmes have resulted in accumulation of smaller genome modifications, e.g. SNPs and INDELs. Consequently, each distinct potato cultivar harbours a unique set of transcripts, resulting in physiological and developmental differences and different responses to biotic and abiotic stress. SNP and INDEL profile differences and novel gene variants in anthocyanin pathway were identified in a comparative transcriptome analysis of two Chinese potato cultivars^9^. Unfortunately, we could not include these transcriptomes in our pan-transcriptome because the assemblies were not publicly accessible.

Based on the DM genome, the PGSC and ITAG annotation consortia^8,10^ have each independently produced potato gene models. For practical reasons, most potato researchers use only one genome annotation, either PGSC or ITAG, especially when conducting high-throughput analyses. Using an incomplete gene set can lead to false conclusions regarding gene presence or gene family diversity in potato. Using a computational pipeline followed by manual curation, we have consolidated the two publicly available Group Phureja DM gene model sets to produce a unified one.

While a combined DM gene set is useful, it is still not as useful as a pan-transcriptome that includes assemblies from cultivated potatoes. However, obtaining an optimal transcriptome from short-read RNA-Seq data is not a trivial task. Each *de novo* assembler suffers from different intrinsic error generation and no single assembler performs best on all datasets^11^. To maximise diversity and completeness of potato cultivar transcriptomes, usage of multiple *de novo* transcriptome assemblers and various parameter combinations over the same input data was employed. Following this”over-assembly” step, we used tr2aacds pipeline from EvidentialGene^12^ to reduce redundancy across assemblies and obtain cultivar-specific transcriptomes. Finally, we consolidated representative cultivar-specific sequences to generate a potato pan-transcriptome (StPanTr). These transcriptomes will improve high throughput sequencing analyses, from RNA-Seq and sRNA-Seq to more specific ones like ATAC-Seq, by providing a more comprehensive and accurate mapping reference. The translated protein sequences can enhance the sensitivity of high-throughput mass-spectroscopy based proteomics. The resource is valuable also for the design of various PCR assays, e.g. quantitative PCR, where exact sequence information is required. Additionally, the knowledge generated regarding variations in transcript sequences between cultivars, such as SNPs, insertions and deletions, will be a key instrument in assisting potato breeding programmes.

## Methods

### Merging PGSC and ITAG gene models of reference genome Group Phureja

**Fig. 1:**
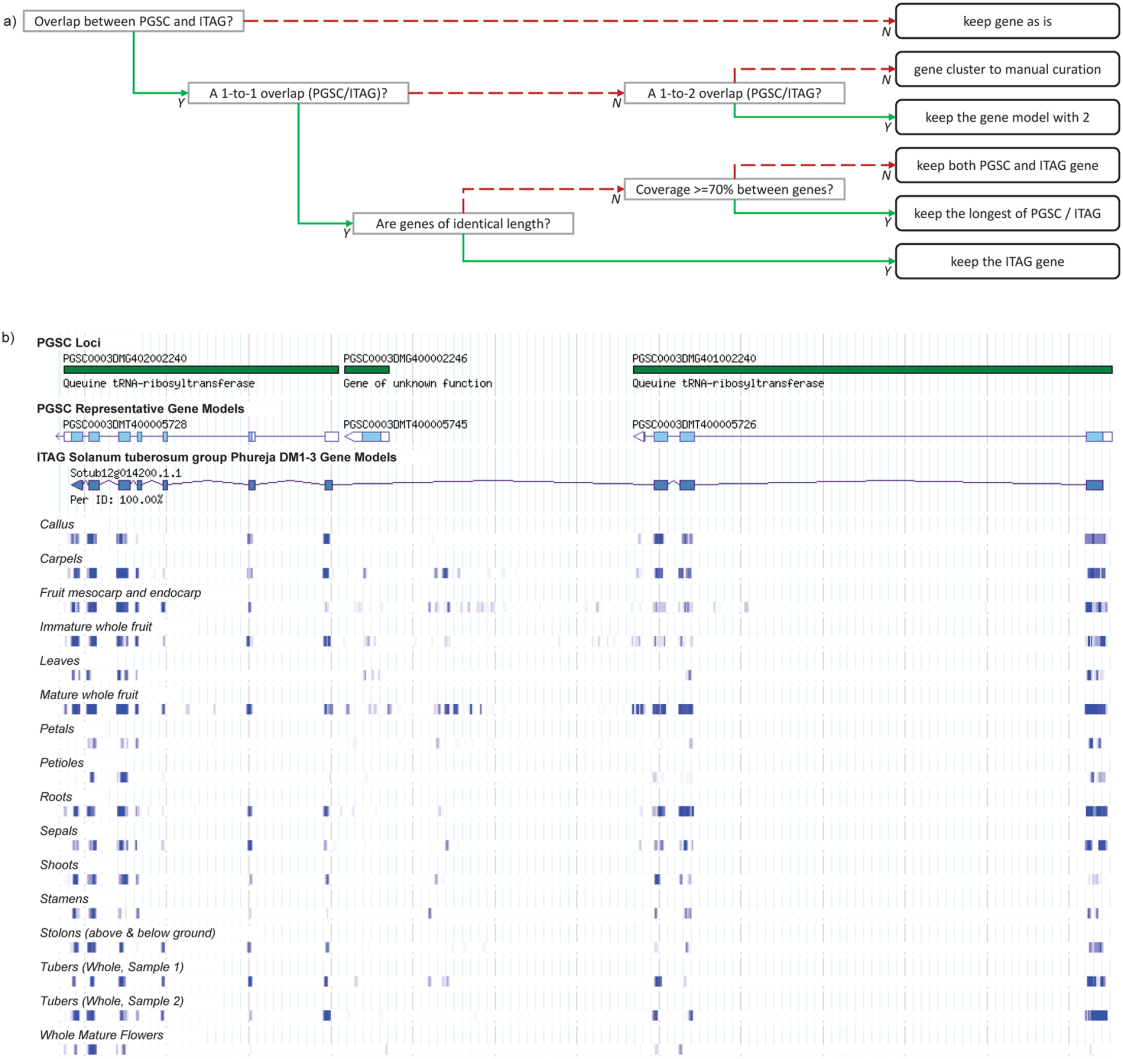
Merging DM Phureja PGSC and ITAG gene models. (**a**) Decision tree for the merging of both genome models, with 6 possible outcomes: singleton genes (‘keep gene as is’), manual curation (‘gene cluster to manual curation’ and programmatic solution (all remaining 4 options). Green solid lines represent a satisfied condition (Y: Yes), dashed red lines, an unsatisfied condition (N: No). (**b**) Example of manual curation in the merged DM genome GTF, region visualisation (chr12:11405699..11418575) in the Spud DB (solanaceae.plantbiology.msu.edu) Genome Browser^13^. ITAG defined Sotub12g014200.1.1 spans three PGSC defined coding sequences (PGSC0003DMT400005728, PGSC0003DMT400005745 and PGSC0003DMT400005726). Below the gene models, RNA sequence tracks are shown, showing how these genes are expressed in various plant organs. In the concrete case, Sotub12g014200.1.1 was preferred due to RNA-Seq evidence being in concordance, and no evidence for PGSC0003DMT400005745.

GFF files corresponding to their respective gene models (PGSC v4.04, ITAG v1.0) were retrieved from the Spud DB (solanaceae.plantbiology.msu.edu) potato genomics resource^13^. The two models (39,431 PGSC and 35,004 ITAG) were then compared on the basis of their exact chromosomal location and orientation in order to create the most complete set of genes from both predicted genome models. Several combinations arose from the merge (Fig. 1a), those for which no action was required (singletons, i.e. sole PGSC or ITAG genes not covering any other genes); 1-to-1 or 1-to-2 combinations between PGSC and ITAG genes, which were solved programmatically; and lastly, combinations of more than 3 genes in various combination types, which continued on to manual curation. The latter (318 gene clusters; example in Fig. 1b) were considered to be non trivial merge examples (overlapping genes in two models or multiple genes in PGSC corresponding to a single gene in ITAG). This resulted in a merged DM genome GFF3 file with 49,322 chromosome position specific sequences, of which 31,442 were assigned with ITAG gene IDs and 17,880 with PGSC gene IDs^14^.

### Data pre-processing

**Fig. 2:**
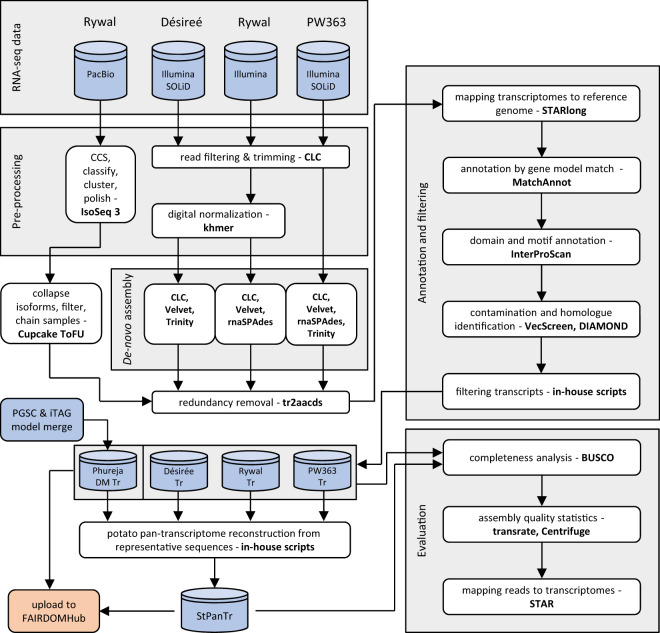
Bioinformatics pipeline for generation of potato transcriptomes. Software used in specific steps are given in bold. Input datasets (sequence reads) and output data (transcriptomes) are depicted as blue cylinders. Data upload steps to public repositories are shaded in orange. Abbreviations: SRA – NCBI Sequence Read Archive, PGSC – Potato Genome Sequencing Consortium, ITAG – international Tomato Annotation Group, CLC – CLC Genomics Workbench, PacBio – Pacific Biosciences Iso-Seq sequencing, Tr – transcriptome, StPanTr – potato pan-transcriptome, tr2aacds – “transcript to amino acid coding sequence” Perl script from EvidentialGene pipeline.

The complete bioinformatic pipeline is outlined in Fig. 2. Sequence quality assessment of raw RNA-Seq data, quality trimming, and removal of adapter sequences and polyA tails was performed using CLC Genomics Workbench v6.5-v10.0.1 (Qiagen) with maximum error probability threshold set to 0.01 (Phred quality score 20) and no ambiguous nucleotides allowed. Minimal trimmed sequence length allowed was set to 15 bp while maximum up to 1 kb. Orphaned reads were re-assigned as single-end (SE) reads. Processed reads were pooled into cultivar datasets as properly paired-end (PE) reads or SE reads per cultivar per sequencing platform. For the Velvet assembler, SOLiD reads were converted into double encoding reads using perl script “denovo_preprocessor_solid_v2.2.1.pl”^15^. To reduce the size of cv. Désirée and cv. Rywal datasets, digital normalization was performed using khmer from bbmap suite v37.68^16^ prior to conducting *de novo* assembly using Velvet and rnaSPAdes.

PacBio long reads were processed for each sample independently using Iso-Seq 3 analysis software (Pacific Biosciences). Briefly, the pipeline included Circular Consensus Sequence (CCS) generation, full-length reads identification (”classify” step), clustering isoforms (”cluster” step) and”polishing” step using Arrow consensus algorithm. Only high-quality full-length PacBio isoforms were used as input for further steps.

### PacBio Cupcake ToFU pipeline

Cupcake ToFU (github.com/Magdoll/cDNA_Cupcake) scripts^17^ were used to further refine the Iso-Seq transcript set. Redundant PacBio isoforms were collapsed with “collapse_isoforms_by_sam.py” and counts were obtained with “get_abundance_post_collapse.py”. Isoforms with less than two supporting counts were filtered using “filter_by_count.py” and 5′-degraded isoforms were filtered using “filter_away_subset.py”. Isoforms from the two samples were combined into one non-redundant Iso-Seq transcript set using “chain_samples.py”.

### De Bruijn graph based *de novo* assembly of short reads

Short reads were *de novo* assembled using Trinity v.r2013-02-25^18^, Velvet/Oases v. 1.2.10^19^, rnaSPAdes v.3.11.1^20^ and CLC Genomics Workbench v8.5.4-v10.1.1 (Qiagen). Illumina and SOLiD reads were assembled separately. For CLC Genomics *de novo* assemblies, combinations of three bubble sizes and 14 k-mer sizes were tested on PW363 Illumina dataset. Varying bubble size length did not influence the assembly statistics much (Supplementary Fig. 2), therefore we decided to use the length of 85 bp for Illumina datasets of the other two cultivars. Bubble size and k-mer length parameters used for Velvet and CLC are given in Table 1. The scaffolding option in CLC and Velvet was disabled. More detailed information per assembly is provided in Auxiliary Table 2^21^.

**Table 1:** Parameters used for short read *de novo* assembly generation.

Genotype	Assembly ID	Read type	Assembler	Assembler version	k-mer length (word size)	Bubble size
Désirée	CLCdnDe8	SOLiD	CLC *de novo*	9.1	24	50
Désirée	CLCdnDe1	SOLiD	CLC *de novo* -	10.0.1	24	50
			- transcript discovery as reference			
Désirée	VdnDe8, …,	SOLiD	Velvet/Oases	1.2.10	23, 33, 43	Default
	…, VdnDe10					
Désirée	CLCdnDe9, …,	Illumina	CLC *de novo*	9.1	21, 23, 33, 43,	85
	…, CLCdnDe14				53, 63	
Désirée	CLCdnDe2, …,	Illumina	CLC *de novo* -	10.0.1	21, 23, 33, 43,	85
	…, CLCdnDe7		- transcript discovery as reference		53, 63	
Désirée	TDe	Illumina	Trinity	r2013-02-25	25	NA
Désirée	VdnDe1, …,	Illumina	Velvet/Oases	1.2.10	23, 33, 43, 53,	Default
	…, VdnDe7				63, 73, 83	
PW363	CLCdnPW1	SOLiD	CLC *de novo*	8.5.4	24	50
PW363	CLCdnPW2	SOLiD	CLC *de novo* -	9.1	24	50
			- transcript discovery as reference			
PW363	VdnPW8, …,	SOLiD	Velvet/Oases	1.2.10	23, 33, 43	Default
	…, VdnPW10					
PW363	CLCdnPW3, …,	Illumina	CLC *de novo*	8.5.4	21, 23, 24, 25, 30,	50, 65, 85
	…, CLCdnPW44				33, 35, 40, 43, 45,	
					50, 53, 55, 63	
PW363	CLCdnPW45, …,	Illumina	CLC *de novo* -	10.0.1	21, 23, 33, 43,	85
	…, CLCdnPW50		- transcript discovery as reference		53, 63	
PW363	SdnPW1	Illumina	rnaSPAdes	3.11.1	43	Default
PW363	TPW	Illumina	Trinity	r2013-02-25	25	NA
PW363	VdnPW1, …,	Illumina	Velvet/Oases	1.2.10	23, 33, 43, 53,	Default
	…, VdnPW7				63, 73, 83	
Rywal	PBdnRY1	PacBio Isoseq	Iso-Seq. 3,	2017	NAp	NAp
			Cupcake ToFU			
Rywal	CLCdnRY1, …,	Illumina	CLC *de novo*	9.1	21, 23, 33, 43,	85
	…, CLCdnRY6				53, 63	
Rywal	CLCdnRY7, …,	Illumina	CLC *de novo* -	10.1.1	21, 23, 33, 43,	85
	…, CLCdnRY12		- transcript discovery as reference		53, 63	
Rywal	SdnRY1	Illumina	rnaSPAdes	3.11.1	43	Default
Rywal	VdnRY1, …,	Illumina	Velvet/Oases	1.2.10	23, 33, 43, 53,	Default
	…, VdnRY7				63, 73, 83	

NAp – not applicable.

NA – not available.

### Decreasing redundancy of assemblies and annotation

739 mio Désirée short reads were assembled into 3,765,661 potential transcripts, 284 mio PW363 short reads were assembled into 6,022,291 potential transcripts, and 710 mio Rywal short reads and 1.4 mio Rywal PacBio sequences were assembled into 1,912,821 potential transcripts. While generation of several transcriptomes from diverse input data and various parameter combinations increases the likelihood of capturing and accurately assembling transcripts^22^, redundancy reduction without loss of information and error removal from the over-assemblies is required. All cultivar-specific transcriptome assemblies, compiled into cultivar-specific transcriptome over-assembly, were initially filtered with the tr2aacds pipeline (part of EvidentialGene v2016.07.11^12^) which consists of four steps. First, all perfect redundant nucleotide sequences are removed using fastnrdb, part of the exonerate package^23^, leaving only the transcript with the longest coding region. Next, all perfect fragments of the remaining transcripts are removed using cd-hit^24^. These first two steps are important in reducing transcriptome redundancy, as true transcripts are expected to be assembled independently by multiple of the assembly methods. Keeping the transcripts with the longest and most complete coding region helps eliminate chimeric and misassembled transcripts, as these errors tend to occur more often in UTR regions or in a manner that causes frameshifts and long, incomplete coding regions^12^).

The third and the fourth step of the tr2aacds pipeline segregate transcripts that are likely isoforms, alleles, or other variations seen at a single locus. This is done through amino acid sequence clustering, which identifies putative transcripts that vary only in silent mutations, and through reciprocal BLAST, which detects high-identity exon-sized alignments (likely isoforms). A tag is assigned to all transcripts providing detailed information on why they were discarded (e.g. perfect fragments, perfect duplicates, very high similarity, …) or why they were marked as alternatives (and which sequence they are an alternative form of). The final output of the tr2aacds pipeline are three sets of data – a non-redundant set of representative sequences (i.e. main set), a set of putative alternatives mapped to the representative set (i.e. alt set), and a discarded set (i.e. drop set) of redundant sequences. It is important to note that not all dropped sequences are of poor quality or incorrect – many of them are dropped due to full or partial redundancy.

Representative and alternative sets (termed okay sets by EvidentialGene) were merged into initial cultivar reference transcriptomes and, as tr2aacds only uses internal evidence for data curation, used in further external evidence for assembly validation, filtering and annotation steps (Fig. 2). The *de novo* cultivar-specific transcript sets were first mapped to the DM reference genome by STARlong 2.6.1d^25^ using parameters optimized for *de novo* transcriptome datasets (all scripts are deposited at FAIRDOMHub (fairdomhub.org) project home page^26^). Aligned transcripts were analysed with MatchAnnot to identify transcripts that match the PGSC or ITAG gene models. Domains were assigned to the polypeptide dataset using InterProScan software package v5.37-71.0^27^. For all transcripts and coding sequences, annotations using DIAMOND v0.9.24.125^28^ were generated by querying UniProt (www.uniprot.org) retrieved databases (E-value cut-off 10^−0.5^ and query transcript/cds and target sequence alignment coverage higher or equal to 50%; custom databases: *Solanum tuberosum*, *Solanaceae*, plants). Initially assembled transcriptomes were also screened for nucleic acid sequences that may be of vector origin (vector segment contamination) using VecScreen plus taxonomy program v.0.16^29^ against NCBI UniVec Database (ftp.ncbi.nlm.nih.gov/pub/UniVec). Potential biological and artificial contamination was identified as up to 3.3% of sequences per cultivar, if taking into account cases when potential contaminants covered less than 1% of the sequence (number of sequences with strong, moderate and weak proof of contamination as follows: 182, 547 and 10,509 for Désirée; 48, 228 and 7,877 for PW363; 169, 179 and 4,103 for Rywal). The results from MatchAnnot, InterProScan and DIAMOND were used as biological evidence in further filtering by in-house R scripts. Transcripts that did not map to the genome nor had any significant hits in either InterPro (www.ebi.ac.uk/interpro) or UniProt (www.uniprot.org) were eliminated from further analysis to obtain higher reliability of constructed transcriptomes^30–32^. Pajek v5.08^33^, in-house scripts, and cdhit-2d from the CD-HIT package v4.6^24^ were used to re-assign post-filtering representative and alternative classes and to obtain finalised cultivar-specific transcriptomes.

The whole redundancy removal procedure reduced the initial transcriptome assemblies by 18-fold for Désirée, 38-fold for Rywal, and 24-fold for PW363. Completeness of each initial *de novo* assembly to cultivar-specific transcriptome was estimated with BUSCO (Supplementary Figs. 1–3).

Individual contributions by various assembly methods were investigated in light of what contributed to the final, cleaned cultivar transcriptomes. SOLiD assemblies (Supplementary Fig. 1: CLCdnDe1, CLCdnDe8, VdnDe8-10), produced by either CLC or Velvet/Oases pipelines, contributed least to transcriptomes, which can mostly be attributed to short length of the input sequences. Interestingly, increasing k-mer size in the CLC pipeline for Illumina assemblies produced more complete assemblies according to BUSCO scores and more transcripts were selected for the initial transcriptome (Supplementary Fig. 1: CLCdnDe1-7, CLCdnDe9-14). On the contrary, increasing k-mer length in Velvet/Oases pipeline lead to transcripts that were less favoured by the redundancy removal procedure (Supplementary Fig. 1: VdnDe1-7). The Trinity assembly was comparable to the high k-mer CLC assemblies in transcriptome contribution and BUSCO score (Supplementary Fig. 1). It might seem that the PacBio Iso-Seq transcripts contributed less than expected to the cv. Rywal transcriptome (Supplementary Fig. 3), however it should be noted that a considerable number of PacBio transcripts was assigned to the EvidentialGene drop set because they had perfect or near-perfect CDS identity of transcripts assembled by CLC. The EvidentialGene pipeline also prioritised CLC-assembled transcripts over PacBio transcripts because the redundancy removal algorithm reorders the near-perfect duplicates by transcript name and only retains the first transcript listed (Auxiliary Table 6^34^).

### Potato pan-transcriptome construction

While the PGSC gene model defined transcripts as well as coding sequences, the ITAG gene model defined only coding sequences. Therefore, the potato pan-transcriptome construction was conducted at the level of CDS.

Cultivar-specific representative coding sequences (57,943 of Désirée, 43,883 of PW363 and 36,336 of Rywal) were combined with coding sequences from the merged Phureja DM gene models (17,880 and 31,442 non-redundant PGSC and ITAG genes, respectively) and subjected to the cdhit-est^24^ algorithm (global sequence identity threshold 90%, alignment coverage for the shorter sequence 75%, bandwidth of alignment 51 nt and word length of 9) to create potato pan-trancriptome. Sequences that did not cluster using cdhit-est were separated into tetraploid and DM datasets and subjected to the cdhit-2d^24^ algorithm (local sequence identity threshold 90%, alignment coverage for the shorter sequence 45%, bandwidth of alignment 45 nt and word length of 5).

**Fig. 3:**
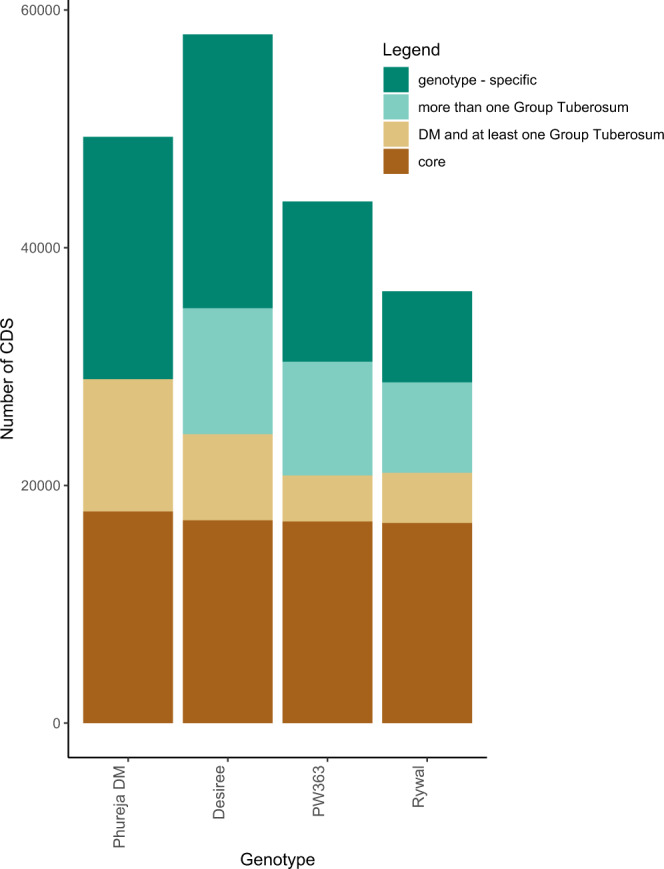
Structure of the potato pan-transcriptome. Stacked bar plot showing the overlap of paralogue groups in cultivar-specific transcriptomes and merged Phureja DM gene model. Only representative and alternative transcripts of the pan-transcriptome are counted (i.e. cultivar representative sequences) while disregarding additional cultivar alternative transcripts. For Phureja DM, the merged ITAG and PGSC DM gene models were counted. DM and at least one Group Tuberosum: sequences shared by Phureja DM and at least one tetraploid genotype, core: sequences shared among all genotypes in the pan-transcriptome.

Sequences that are shared by the DM merged gene model and *de novo* assembled cultivar-specific transcriptomes were designated as “core” transcripts, and sequences that were assembled in only one transcriptome were designated “genotype-specific”. The total pan-transcriptome includes 96,886 representative, non-redundant transcripts and 90,618 alternative sequences (covering alternative splice forms, allelic isoforms and partial transcripts) for those loci (Fig. 3, Supplementary Fig. 4, Auxiliary Table 7^35^). The core subset of the pan-transcriptome contains 68,708 sequences, among which 12% are partial sequences.

Polyploid crop pan-genomes generally consist of many cultivar-specific genes^36^. Therefore we included all genotype-specific sequences in our potato pan-transcriptome (Fig. 3, Supplementary Fig. 4). This subset contains 64,529 sequences, among which 13% sequences are partial^35^. Genotype-specific transcripts are generally shorter in length than the core transcripts, however they do not differ much in the percentage of complete transcripts.

## Data records

Transcriptomic sequences of three potato genotypes, cv. Désirée, cv. Rywal and breeding clone PW363, were obtained from in-house RNA-Seq projects^37–41^ and supplemented by publicly available cv. Désirée datasets retrieved from SRA^42–44^ (Table 2). All generated files have been deposited to FAIRDOMhub^26^ under project name _p_stRT (https://fairdomhub.org/projects/161).

**Table 2:** Samples used to generate the *de novo* transcriptome assemblies.

Genotype	Sample description^a^	Sequencing platform	Library type^b^	Number of reads^c^	SRA ID
Désirée	PVY inoculated leaves	Illumina	DSN-normalized	~54 mio	SRR10070125
			PE90 unstranded		
Désirée	non-transformed and PVY-inoculated plants, non-infested and CPB infested leaves	Illumina	PE90 unstranded	~195 mio	SRR1207287, …, SRR1207290
Désirée	mock and PVY inoculated leaves and stem	SOLiD	SE50 unstranded	~154 mio	SRR10065428, SRR10065429
Désirée	leaves	Illumina	SE50 unstranded	~172 mio	SRR3161991, SRR3161995, SRR3161999, SRR3162003, SRR3162007, SRR3162011, SRR3162015, SRR3162019, SRR3162023, SRR3162027, SRR3162031, SRR3162035
Désirée	seedlings	Illumina	SE100 unstranded	~80 mio	SRR4125238, …, SRR4125247
Désirée	roots	Illumina	SE100 unstranded	~31 mio	SRR4125248, …, SRR4125252
Désirée	mock and *Phytophthora infestans* inoculated leaves	Illumina	PE90 unstranded	~53 mio	ERR305632
Rywal	mock and PVY inoculated leaves	PacBio	Iso-Seq, 0.7–2 Kb, 2–3.5 Kb, >3.5 Kb	~1.4 mio	SRR8281993, …, SRR8282008
				CCS	
Rywal	mock and PVY inoculated leaves	Illumina	PE100 strand-specific	~710 mio	SRX6801457, …, SRX6801468
PW363	PVY inoculated leaves	Illumina	DSN-normalized	~104 mio	SRR10070123, SRR10070124
			PE90 unstranded		
PW363	mock and PVY inoculated leaves	SOLiD	SE50 unstranded	~180 mio	SRR10065430, …, SRR10065433

^a^PVY, *Potato virus Y*; CPB, Colorado potato beetle.

^b^PE, paired-end library (the number stands for read length in nt); SE, single-end library (the number stands for read length in nt); DSN-normalized, RNA-Seq library utilizing the crab duplex nuclease; CCS, circular consensus sequences.

^c^For paired-end libraries, pairs are counted as two reads.

The largest quantity of reads, cca. 739 mio reads of various lengths, was obtained for cv. Désirée, using Illumina and SOLiD short-read sequencing platforms. For cv. Rywal and breeding clone PW363 only mature leaf samples were available. For cv. Désirée leaf samples were augmented with samples from stems, seedlings and roots. For cv. Rywal short-read sequencing was complemented with full-length PacBio Iso-Seq sequencing of independent samples. Detailed sample information is provided in Auxiliary Table 1^45^.

Cv. Rywal NPR1-1 coding sequences, sequenced by the Sanger method, were deposited at NCBI GenBank under accession numbers MT210578, …, MT210585^46–53^.

The GTF file with merged ITAG and PGSC gene models for *S*. *tuberosum* Group Phureja DM genome v4.04^14^ is also available at FAIRDOMHub project page, as well as the cultivar-specific and pan-transcriptome assembly FASTA and annotation files^54^.

## Technical Validation

As a measure of assembly accuracy, the percentage of correctly assembled bases was obtained by mapping Illumina reads back to cultivar-specific initial transcripts using STAR v.2.6.1d RNA-seq aligner with default parameters (Table 3). To assess the quality of transcriptomes via size-based and reference-based metrics, we ran TransRate v 1.0.1^55^ on cultivar-specific transcriptomes (prior to and post filtering, Table 4), cultivar-specific representative transcript sequences and PGSC defined representative transcripts (Table 5). Comparative metrics for cultivar-specific coding sequences (CDS) were obtained using Conditional Reciprocal Best BLAST (CRBB)^56^ against merged Phureja DM gene model coding sequences.

**Table 3:** Transcriptome quality control by RNA-seq reads remapping.

Mapping statistics/genotype	Désirée’	PW363’	Rywal’
Number of input reads	177,149,132	52,171,015	342,767,035
Average input read length	178	179	199
**UNIQUE READS:**			
Uniquely mapped reads number	64,507,790	18,416,487	206,003,021
**Uniquely mapped reads**^**#**^**%**	**36%**	**35%**	**60%**
Average mapped length	175	176	196
Number of splices*: Total	496,170	267,268	1,700,235
Number of splices*: Annotated (sjdb)	0	0	0
Number of splices*: GT/AG	258,208	105,551	1,162,885
Number of splices*: GC/AG	10,749	5,693	79,495
Number of splices*: AT/AC	1,486	2,192	1,840
Number of splices*: Non-canonical	225,727	153,832	456,015
Mismatch rate per base %	0.50%	0.53%	0.59%
Deletion rate per base	0.03%	0.03%	0.03%
Deletion average length	2.72	2.53	3.02
Insertion rate per base	0.02%	0.02%	0.03%
Insertion average length	1.93	1.86	1.91
**MULTI-MAPPING READS:**			
Number of reads mapped to multiple loci	98,694,222	29,366,122	108,669,657
**% of reads mapped to multiple loci**^**#**^	**56%**	**56%**	**32%**
Number of reads mapped to too many loci	4,652,918	1,555,704	1,541,238
% of reads mapped to too many loci	2.63%	2.98%	0.45%
**UNMAPPED READS:**			
% of reads unmapped: too many mismatches	0%	0%	0%
% of reads unmapped: too short	5.25%	5.43%	7.75%
% of reads unmapped: other	0%	0%	0%
**CHIMERIC READS:**			
Number of chimeric reads	0	0	0
% of chimeric reads	0%	0%	0%

Illumina paired-end reads used for generating assemblies were mapped back to the corresponding cultivar specific transcriptomes using STAR.

*Number of reads crossing supposed splice sites.

‘Initially constructed transcriptomes (prior to filtering steps).

^#^Relevant % of mapped reads: % of uniquely mapped reads + % of reads mapped to multiple loci.

**Table 4:** Prior and post-filtering transcriptome summary statistics for potato cultivar-specific coding sequences generated by TransRate.

TransRate metrics	Désirée		PW363		Rywal	
	Pre-filter (initial)	Post-filter	Pre-filter (initial)	Post-filter	Pre-filter (initial)	Post-filter
**CONTIG METRICS**						
No. sequences	350,271	197,839	273,216	159,278	134,755	79,095
Sequence mean length	504	792	516	775	459	707
No. sequences under 200 nt	125,465	25,330	88,230	17,370	52,653	13,198
No. sequences over 1000 nt	57,679	55,837	44,508	42,571	19,175	18,748
No. sequences over 10000 nt	23	23	3	3	1	1
’n90	369	444	366	429	351	390
’n50	1,194	1,209	1,110	1,131	1,227	1,218
GC %	41%	42%	42%	42%	42%	42%
Ambiguous nucleotide (N) %	0%	0%	0%	0%	0%	0%
**COMPARATIVE METRICS**						
No. seq. with CRBB hits*	160,295	138,131	138,443	116,834	66,258	55,239
No. reference seq. with CRBB hits*	29,858	27,642	25,739	23,839	23,549	22,163
coverage50^#^*	25,991	24,586	21,875	20,620	20,258	19,538
coverage95^#^*	19,329	18,246	15,664	14,727	14,967	14,470
Reference coverage*	65%	63%	56%	54%	53%	52%

’The largest contig size at which at least 90% or 50% of bases are contained in contigs at least this length.

*Reference-based summary statistics (merged Phureja DM coding sequences were used as reference).

^#^Proportion of reference proteins with at least N% of their bases covered by a Conditional Reciprocal Best Blast (CRBB) hit.

**Table 5:** Summary statistics for potato cultivar-specific representative transcript sequences generated by TransRate.

TransRate metrics	Désirée	PW363	Rywal	PGSC^+^
**CONTIG METRICS**				
No. sequences	57,943	43,883	36,336	39,031
Sequence mean length	922	926	1,028	1,283
No. sequences under 200 nt	875	1,377	1,310	87
No. sequences over 1000 nt	18,500	14,545	14,307	20,226
No. sequences over 10000 nt	13	6	2	0
’n90	369	387	440	645
’n50	1,566	1,535	1,673	1,726
GC %	40%	41%	41%	40%
Ambiguous nucleotide (N) %	0%	0%	0%	0%
**COMPARATIVE METRICS**				
No. seq. with CRBB hits*	38,034	30,826	28,389	38,600
No. reference seq. with CRBB hits*	25,094	21,751	21,299	37,534
coverage50^#^*	12,799	10,693	7,909	36,379
coverage95^#^*	8,053	6,430	5,053	30,187
Reference coverage*	33%	28%	20%	75%

’The largest contig size at which at least 90% or 50% of bases are contained in contigs at least this length.

*Reference-based summary statistics (merged Phureja DM coding sequences were used as reference).

^#^Proportion of reference proteins with at least N% of their bases covered by a Conditional Reciprocal Best Blast (CRBB) hit.

^+^PGSC_DM_v3.4_transcript-update_representative.fasta.zip file from Spud DB was used for Phureja-specific representative transcript sequences (PGSC).

To estimate the measure of completeness and define the duplicated fraction of assembled transcriptomes (prior and post filtering cultivar-specific transcriptomes and pan-transcriptome), BUSCO v3^57^ scores were calculated using embryophyta_odb9^58^ lineage data (Table 6). At the cultivar-specific transcriptome level, the most diverse dataset in terms of tissues and experimental conditions, the cv. Désirée dataset, resulted in the highest BUSCO score as expected. The success in classification of representative and alternative transcripts is evident from the pan-transcriptome BUSCO scores (i.e. differences in single-copy and duplicated BUSCOs for representative and alternative dataset). The highest number of fragmented BUSCOs is observed for the breeding clone PW363, which we can probably attribute to the highest number of short-contig assemblies. Furthermore, the long-read assembly presumably contributed to the shift in favour of single-copy BUSCOs (Table 6) and uniquely mapped reads (Table 3) for cv. Rywal.

**Table 6:** Assessment of completeness of constructed transcriptomes.

cv. Désirée	initial rep+alt	post 1st filtering rep+alt	final rep+alt
(**S**)	37.8	37.8	37.4
(**D**)	59.4	59.2	58.4
(**C**)	**97**.**2**	**97**.**0**	**95**.**8**
(F)	1.1	1.2	1.4
(M)	1.7	1.8	2.8
**breeding clone PW363**	**initial rep+alt**	**post 1st filtering rep+alt**	**final rep+alt**
(**S**)	39.9	39.2	38.4
(**D**)	51.7	51.2	50.9
(**C**)	**91**.**6**	**90**.**4**	**89**.**3**
(F)	2.9	3.4	3.5
(M)	5.6	6.2	7.2
**cv. Rywal**	**initial rep+alt**	**post 1st filtering rep+alt**	**final rep+alt**
(**S**)	55.8	55.8	55.1
(**D**)	35.2	34.8	34.7
(**C**)	**91**.**0**	**90**.**6**	**89**.**8**
(F)	2.4	2.6	2.7
(M)	6.5	6.9	7.5
**pan-transcriptome**	**rep**	**alt**	**rep+alt**
(**S**)	92.2	11.0	3.9
(**D**)	6.1	85.9	95.6
(**C**)	**98**.**3**	**96**.**9**	**99**.**4**
(F)	1.4	1.3	0.3
(M)	0.3	1.7	0.3

Percentage of BUSCOs identified in each transcriptome assembly step.

(S): Complete and single-copy BUSCOs %;

(D): Complete and duplicated BUSCOs %

(C): Complete BUSCOs (S + D) %

(F): Fragmented BUSCOs %

(M): Missing BUSCOs %

rep: representative

alt: alternative

*Database size: 1440.

To inspect the quality of paralogue cluster assignments, multiple sequence alignments (MSA) using MAFFT v7.271^59^ were conducted on representative and alternative sequences from paralogue clusters^35^ containing sequences from each of the four genotypes (Désirée, PW363, Rywal and DM). Alignments were visualized using MView v1.66^60^ (Auxiliary File 2^61^). These alignments were used to quality check *de novo* transcriptome assemblies and helped us optimise the pipeline. The alignments within groups showed differences that can be attributed to biological diversity, e.g. SNPs and INDELS as well as alternative splicing^61^.

To estimate the proportion of transcripts originating from organisms other than potato, we performed a taxonomic classification of all cultivar-specific transcriptome sequences using Centrifuge v1.0.4^62^ and the NCBI nt database. We used pavian^63^ to generate classification summary reports (Auxiliary File 3^64^). The transcriptomes include altogether less than 1% bacterial, viral, fungal and protozoan transcripts. Other non-plant sequences are from common plant pests such the arachnid *Tetranychus urticae* in PW363 transcriptome (cca. 4%) and insects *Leptinotarsa decemlineata* (cca. 0.1%) and *Myzus persicae* (cca. 0.1%) in the Désirée transcriptome^64^.

### Sanger sequencing confirmation of assembled transcripts

To further validate the quality of the constructed Rywal reference transcriptome, the cultivar-specific assembled transcript coding for NPR1-1 protein in cv. Rywal was compared to sequences amplified from isolated cDNA and sequenced by Sanger method. Total RNA was isolated from 4-week-old cv. Rywal plants using RNeasy Plant Mini kit (Qiagen). Residual genomic DNA was digested with DNase I (Qiagen). Treated total RNA was reversed transcribed using High Capacity cDNA Reverse Transcription Kit (Applied Biosystems). The full-length CDS of NPR1-1 (Sotub07g016890.1.1, ITAG genome annotation) was amplified from the cv. Rywal cDNA with forward (5′-ATGGAGAGCGGCCACGAGA-3′) and reverse (5′-CTACTTTTTTCTAAACTTGTGACTGACATT-3′) primers. The PCR product was inserted into pJET1.2/blunt vector with CloneJET PCR Cloning Kit (Thermo Scientific) and introduced into OneShot TOP10 Chemically Competent *Escherichia coli* cells. The plasmids were isolated from 8 transformed colonies, grown on selection media, using the GenElute Plasmid Miniprep Kit (Sigma-Aldrich). Inserts were sequenced (GATC Services, Eurofins Genomics) using pJET1.2 sequencing primers (Thermo Scientific) as well as forward (5′-CTCCAAGGTTGTGAACGAGGTACTT-3′) and reverse (5′-AAGTACCTCGTTCACAACCTTGGAG-3′) insert-specific primers, designed to ensure full sequence coverage in both directions.

**Fig. 4:**
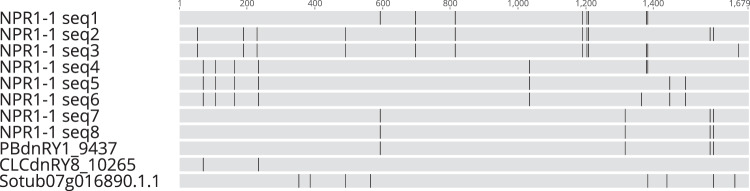
Sanger sequencing validates the constructed cultivar specific transcriptome. Multiple sequence alignment of NPR1-1 coding sequence obtained from eight *E*. *coli* colonies (NPR1-1 seq. 1–8) by the Sanger method, assembled short or long-read cv. Rywal transcripts and Phureja DM gene model (Sotub07g016890.1.1). Grey - sequence identity, black - SNPs. The alignment was prepared and visualised with Geneious Prime 2020.1.1^65^.

A multiple sequence alignment comparing Sanger sequences with NPR1-1 coding sequence from the assembled cv. Rywal transcriptome (paralogue cluster stCuSTr-R_29366) and Phureja DM gene model (Sotub07g016890.1.1) was constructed using Geneious Prime 2020.1.1 (https://www.geneious.com)^65^. The Sanger sequencing revealed the presence of several distinct gene variants in the analysed colonies, differing in the SNP pattern. The PBdnRY1_9437 sequence is validated by complete sequence identity with the two colonies (seq. 7 and 8), while the CLCdnRY_10265 shares two SNPs with seq. 4–6, matching the SNP pattern partially. Although the Phureja DM gene model also shares two SNPs with some of the colonies, its overall SNP pattern differs significantly from cv. Rywal, distinguishing the cultivar-specific transcripts from that of Phureja DM (Fig. 4).

## Usage Notes

### Insights into variability of potato transcriptomes

Based on the comparison of cultivar-specific transcriptomes we identified cca. 23,000, 13,000, and 7,500 paralogue groups of transcripts in cv. Désirée, breeding clone PW363 and cv. Rywal, respectively, not present in the merged Phureja DM gene model. The addition of Iso-Seq dataset in the case of cv. Rywal confirms that long reads contribute to less fragmentation of the *de novo* transcriptome. It is therefore recommended to generate at least a subset of data with one of the long-read technologies to complement the short-read RNA-seq. As it can be seen by the reduction rate in PW363 (24-fold), producing additional short-read assemblies does not contribute to the transcriptome quality as much as having several tissues or a combination of 2^*nd*^ and 3^*rd*^ generation sequencing (38-fold Rywal).

**Fig. 5:**
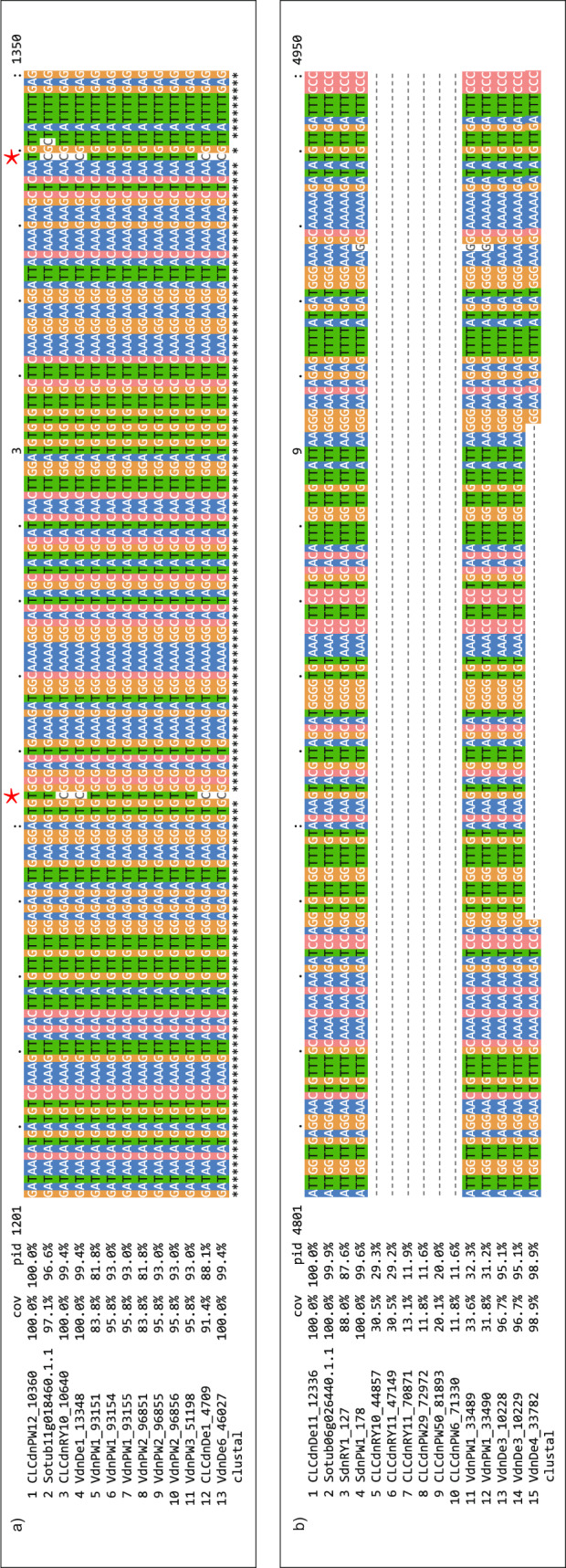
Transcript variants present in pan-transcriptome paralogue gene groups. a) Alignment part of stPanTr_010101 with two PW363-specific SNPs marked by red dots. Such SNPs can be used to design cultivar- or allele-specific qPCR assays. b) Alignment part of stPanTr_074336 showing an alternative splice variant in Désirée, (VdnDe4_33782). Both multiple sequence alignments were made using ClustalOmega v 1.2.1^69^ and visualized with MView v 1.66^60^. The remaining alignments can be found in Auxiliary file 2^61^.

From all four genotypes, cv. Désirée has the highest number of cultivar-specific representative transcripts, which can be attributed to having the most diverse input dataset used for the *de novo* assemblies in terms of tissues sequenced (stem, seedlings and roots) and experimental conditions covered. Cv. Désirée also benefited from the inclusion of a DSN Illumina library to capture low level expressed transcripts. However, even the leaf-specific reference transcriptomes of cv. Rywal and breeding clone PW363 include thousands of specific genes, indicating that cultivar specific gene content is common. Remarkably, we identified several interesting features when inspecting paralogue groups of transcripts, demonstrating the variability of sequences in potato haplotypes and the presence of the alternative splicing variants that contribute to the pan-transcriptome (Fig. 5, Auxiliary File 2^61^).

It should be noted, that the reconstructed transcriptomes include also the meta-transcriptome stemming from microbial communities present in sampled potato tissues (Auxiliary File 3^64^). We decided not to apply any filter on these transcripts. Inclusion of meta-transcripts makes it possible to also investigate the diversity of plant-associated endo- and epiphytes. The majority of these microbial transcripts will have microbial annotations, facilitating their future removal when necessary for other experiments.

### Cultivar-specific transcriptomes can improve high-throughput sequencing analyses

Most gene expression studies have been based on either potato UniGenes, assembled from a variety of potato expressed sequence tags (e.g. StGI, POCI), or the reference DM genome transcript models. Studies based on any of these resources have provided useful information on potato gene expression, but also have major drawbacks.

When using the DM genome as a reference for mapping RNA-Seq reads, the potato research community faces the existence of two overlapping, but not identical, gene model predictions. When using either of available GFFs, we are missing some of the genes known to be encoded in the assembled scaffold. The newly generated merged DM-based GTF helps to circumvent this problem, but even in the merged GTF the cultivar-specific genes and variations are not considered. Differences in expression and important marker transcripts can therefore be missed. In addition, the computational prediction of DM transcript isoforms is incomplete and, in some cases, gene models are incorrectly predicted. On the other hand, the inherent heterogeneity and redundancy of UniGenes or similarly combined transcript sets causes the mapping of short reads to multiple transcripts, thus making the result interpretation more difficult. The cultivar-specific transcriptomes presented here are an improvement, as they include several transcripts missing in the Phureja DM transcriptome. The transcriptomes are also a valuable asset for other high-throughput sequencing applications, such as sRNA-Seq, Degradome-Seq or ATAC-Seq, as we now have more detailed information also on transcript variability within one locus.

The benefit of using cultivar-specific transcriptomes was demonstrated through mapping statistics for Désirée leaf samples under drought stress^66^ to Désirée, ITAG/PGSC merged and PGSC representative transcriptome sequences. Taking all three samples together, 5.5% more reads were uniquely mapped to Désirée than the ITAG/PGSC Phureja DM gene models (Table 7). From the reads mapped to Désirée transcriptome, 5.3% mapped to Désirée-specific transcripts and 8.2% to transcripts specific to Group Tuberosum genotypes (Auxiliary Table 8^67^).

**Table 7:** Mapping of independent dataset to newly assembled cultivar specific reference transcriptome.

Reference	Désirée	ITAG/PGSC	PGSC	Désirée	ITAG/PGSC	PGSC
**Mapping statistics/Sample**	**SRR10416847**			**SRR10416848**		
Number of input reads	14,953,659			14,610,172		
Average input read length	252			252		
**UNIQUE READS:**						
**Uniquely mapped reads**^**#**^**%**	**74%**	**69%**	**71%**	**73%**	**67%**	**69%**
Average mapped length	246	244	246	246	244	246
Number of splices: Total	123,490	104,658	159,842	127,414	110,423	165,595
Number of splices: Non-canonical	40,960	43,764	45,750	40,898	45,459	42,978
Mismatch rate per base %	0.75%	0.94%	1.00%	0.74%	0.93%	1.00%
Deletion rate per base	0.05%	0.04%	0.06%	0.05%	0.04%	0.06%
Deletion average length	3.42	3.46	2.88	3.41	3.47	2.89
Insertion rate per base	0.03%	0.02%	0.04%	0.03%	0.02%	0.04%
Insertion average length	2.21	2.99	2.52	2.24	3.00	2.53
**MULTI-MAPPING READS:**						
**% of reads mapped to multiple loci**^**#**^	**4**.**94%**	**1**.**95%**	**2**.**74%**	**4**.**86%**	**1**.**68%**	**2**.**52%**
% of reads mapped to too many loci	0%	0%	0%	0%	0%	0%
**UNMAPPED READS:**						
% of reads unmapped: too many mismatches	0%	0%	0%	0%	0%	0%
% of reads unmapped: too short	21%	29%	27%	22%	31%	29%
% of reads unmapped: other	0%	0%	0%	0%	0%	0%
**Mapping statistics/Sample**	**SRR10416849**			**all samples**		
Number of input reads	14,755,430			44,319,261		
Average input read length	252			252		
**UNIQUE READS:**						
**Uniquely mapped reads**^**#**^**%**	**49%**	**44%**	**46%**	**66%**	**60%**	**62%**
Average mapped length	245	243	246	246	244	246
Number of splices: Total	95,409	77,103	115,083	346,313	292,184	440,520
Number of splices: Non-canonical	33,269	31,224	31,065	115,127	120,447	119,793
Mismatch rate per base %	0.75%	0.94%	1.01%	0.75%	0.94%	1.00%
Deletion rate per base	0.06%	0.05%	0.07%	0.05%	0.05%	0.06%
Deletion average length	3.23	3.25	2.83	3.36	3.41	2.87
Insertion rate per base	0.03%	0.03%	0.04%	0.03%	0.02%	0.04%
Insertion average length	2.27	3.15	2.56	2.24	3.04	2.54
**MULTI-MAPPING READS:**						
**% of reads mapped to multiple loci**^**#**^	**3**.**60%**	**1**.**19%**	**1**.**94%**	**4**.**47%**	**1**.**61%**	**2**.**40%**
% of reads mapped to too many loci	0%	0%	0%	0%	0%	0%
**UNMAPPED READS:**						
% of reads unmapped: too many mismatches	0%	0%	0%	0%	0%	0%
% of reads unmapped: too short	47%	55%	52%	30%	38%	36%
% of reads unmapped: other	0%	0%	0%	0%	0%	0%

Mapping statistics for Désirée leaf samples under drought stress to Désirée, ITAG/PGSC merged and PGSC representative transcriptome sequences is shown.

RNA-seq data from Désirée leaf samples under drought stress retrieved from the GEO Series GSE140083 – “Transcriptome profiles of contrasting potato (*Solanum tuberosum L*.) genotypes under water stress”. No chimeric reads detected.

^#^Relevant % of mapped reads: % of uniquely mapped reads + % of reads mapped to multiple loci.

Cultivar-specific transcriptomes may also help improve mass-spectroscopy based proteomics. Comprehensive protein databases, obtained from transcriptomic data, offer a higher chance of finding significant targets with peptide spectrum match algorithms, thus enhancing the detection and sensitivity of protein abundance measurements^68^.

### Using transcriptomes to inform qPCR amplicon design

Aligning transcript coding sequences from a StPanTr paralogue cluster can be used to inform qPCR primer design in order to study gene expression in different cultivars or specific isoforms by selecting variable transcript regions (Fig. 5). On the other hand, when qPCR assays need to cover multiple cultivars, the nucleotide alignments can be inspected for conservative regions for design.

### Limitations

Although the here presented transcriptomes are of higher quality than assemblies produced by a single assembler they are neither comprehensive nor perfectly accurate. Firstly, for cv. Rywal and breeding clone PW363 reference transcriptomes are leaf-specific. Additionally, some transcripts are represented by partial sequence(s) and some might be misassembled. Due to very low expression, some transcripts are missed. One also has to note that some true transcripts with very high similarity to the representative transcript, potentially expressed from distinct alleles differing in the SNP pattern, were removed by filtering. Furthermore, most long non-coding mRNAs are discarded in the EvidentialGene step.

When mapping reads back to the initial transcriptomes a fraction of reads was spliced (Table 3), indicating that the transcripts to which these reads map might originate from splice variants that are not present in the transcriptome. We would however like to point out that contiguity of this reads was not confirmed thus they might represent misassemblies. In Désirée 14,094 transcripts had uniquely mapped spliced reads (7% of the filtered transcriptome), and similarly 7,493 (9%) transcripts in Rywal and 10,220 (6%) in PW363. Low number of non-canonical sites was however confirmed by STARlong mapping: 118 for Désirée, 59 for Rywal and 101 for PW363.

An indication of potential problems with some of the assemblies is that the number of transcripts with length less than 200 nt is 10x higher in constructed cultivar-specific transcriptomes than in DM Phureja PGSC representative transcripts (875, 1377 and 1310 vs. 87 for Désirée, PW363 and Rywal vs. DM Phureja) while the transcript length is about 300 nt shorter in average (Table 5). To check for any misassemblies for genes of interest the users are advised to check the MSA of the paralogue cluster (examples for core transcriptome subset can be found in Auxiliary File 2^61^). Some of the assembled sequences are also not of potato origin. Vecscreen implied vector segment contamination in 46 Désirée, 9 PW363 and 31 Rywal sequences, while Centrifuge classified some sequences as originating from common plant pests, among which are the meta-transcriptome sequences of potato microbial communities and the complete genome of the Potato Virus Y (PVY), present due to experimental treatments.

## Supplementary information

Supplementary Information

## Data Availability

All used Bash, Perl, Python, and R/Markdown custom code and scripts complemented with intermediate and processed data (input and output files), and all other supporting information that enable reproduction and re-use are available at FAIRDOMHub under project name _p_stRT (fairdomhub.org/projects/161) under CC BY 4.0 licenses. Data were locally stored in a ISA-tab compliant project directory tree generated using pISA-tree (github.com/NIB-SI/pISA) and uploaded to FAIRDOMHub repository using FAIRDOMHub API and R package pisar (github.com/NIB-SI/pisar).
