# Supplementary material for: Cultivar-specific transcriptome and pan-transcriptome reconstruction of tetraploid potato
Source: Sci Data. 2020 Jul 24;7:249. doi: 10.1038/s41597-020-00581-4 (PMC7382494; doi:10.1038/s41597-020-00581-4)

Supplementary Information

Supplementary Figures

Supplementary Figure 1. Contribution of individual *de novo* assemblies to cultivar Désirée transcriptome. Proportion of all contigs in de novo assembly (blue bars) and proportion of EvidentialGene okay set (green bars), and the number of complete BUSCOs (dots) using embryophyta_odb9 set are shown. Assembly software abbreviations: CLCdn - CLC Genomics Workbench, Vdn - Velvet. http://doi.org/10.15490/FAIRDOMHUB.1.DATAFILE.3107.1

Supplementary Figure 2. Contribution of individual *de novo* assemblies to cultivar PW363 transcriptome. Proportion of all contigs in de novo assembly (blue bars) and proportion of EvidentialGene okay set (green bars), and the number of complete BUSCOs (dots) using embryophyta_odb9 set are shown. Assembly software abbreviations: CLCdn - CLC Genomics Workbench, Vdn - Velvet, Sdn - SPAdes. http://doi.org/10.15490/FAIRDOMHUB.1.DATAFILE.3108.1

Supplementary Figure 3. Contribution of individual *de novo* assemblies to cultivar Rywal transcriptome. Proportion of all contigs in de novo assembly (blue bars) and proportion of EvidentialGene okay set (green bars), and the number of complete BUSCOs (dots) using embryophyta_odb9 set are shown. Assembly software abbreviations: CLCdn - CLC Genomics Workbench, Vdn - Velvet, Sdn - SPAdes, PBdn - PacBio. http://doi.org/10.15490/FAIRDOMHUB.1.DATAFILE.3109.1

Supplementary Figure 4. Venn diagrams showing the overlap of paralogue clusters in cultivar-specific transcriptomes and merged Phureja DM gene models. Representatives and alternatives of the stPanTr (pan-transcriptome) paralogue cluster are counted as well as alternatives defined at stCuSTr (cultivar-specific transcriptome) step. For Phureja, the merged ITAG and PGSC DM gene models were counted. http://doi.org/10.15490/FAIRDOMHUB.1.DATAFILE.3409.2

Supplementary Figure 1.

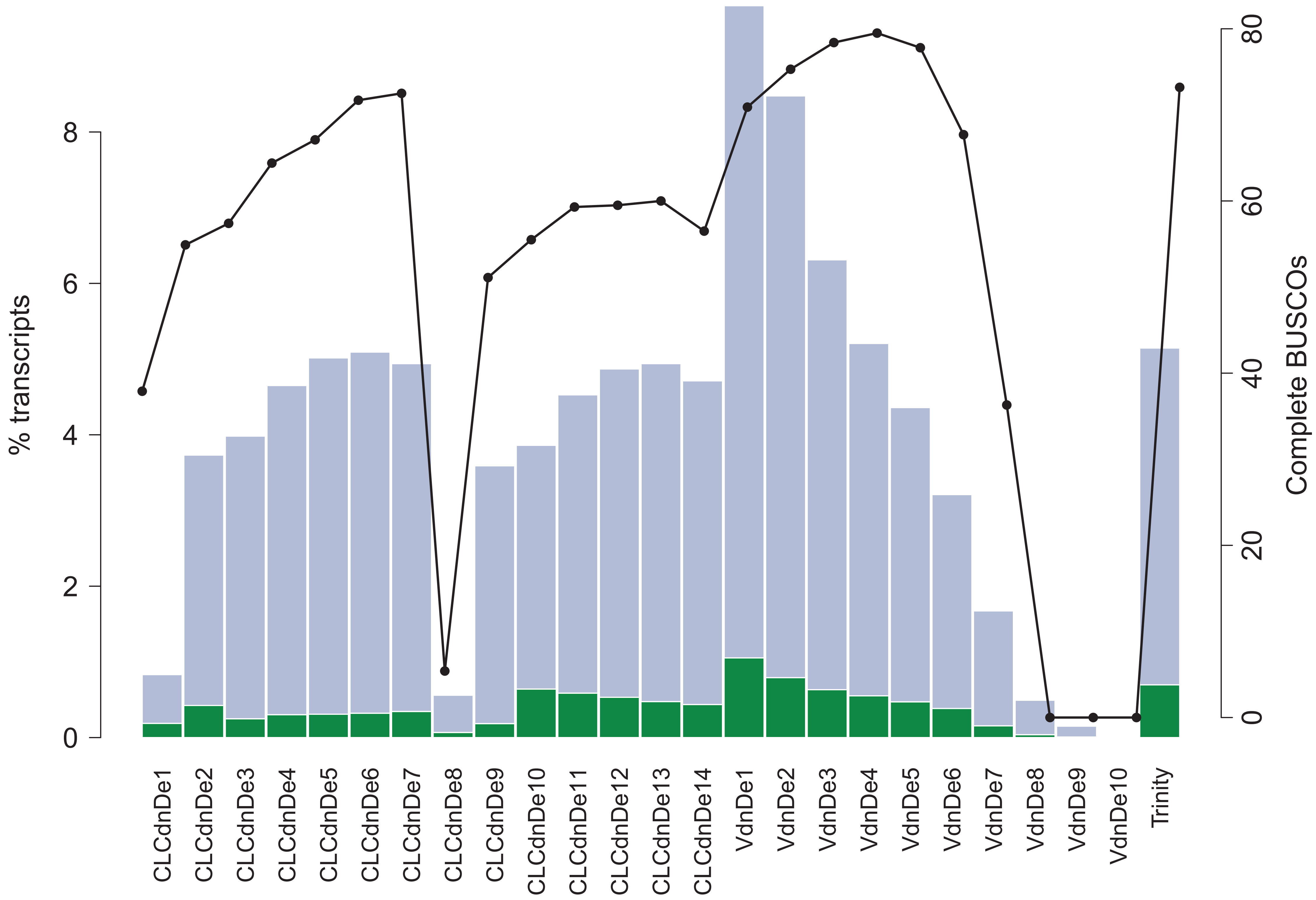

Supplementary Figure 2.

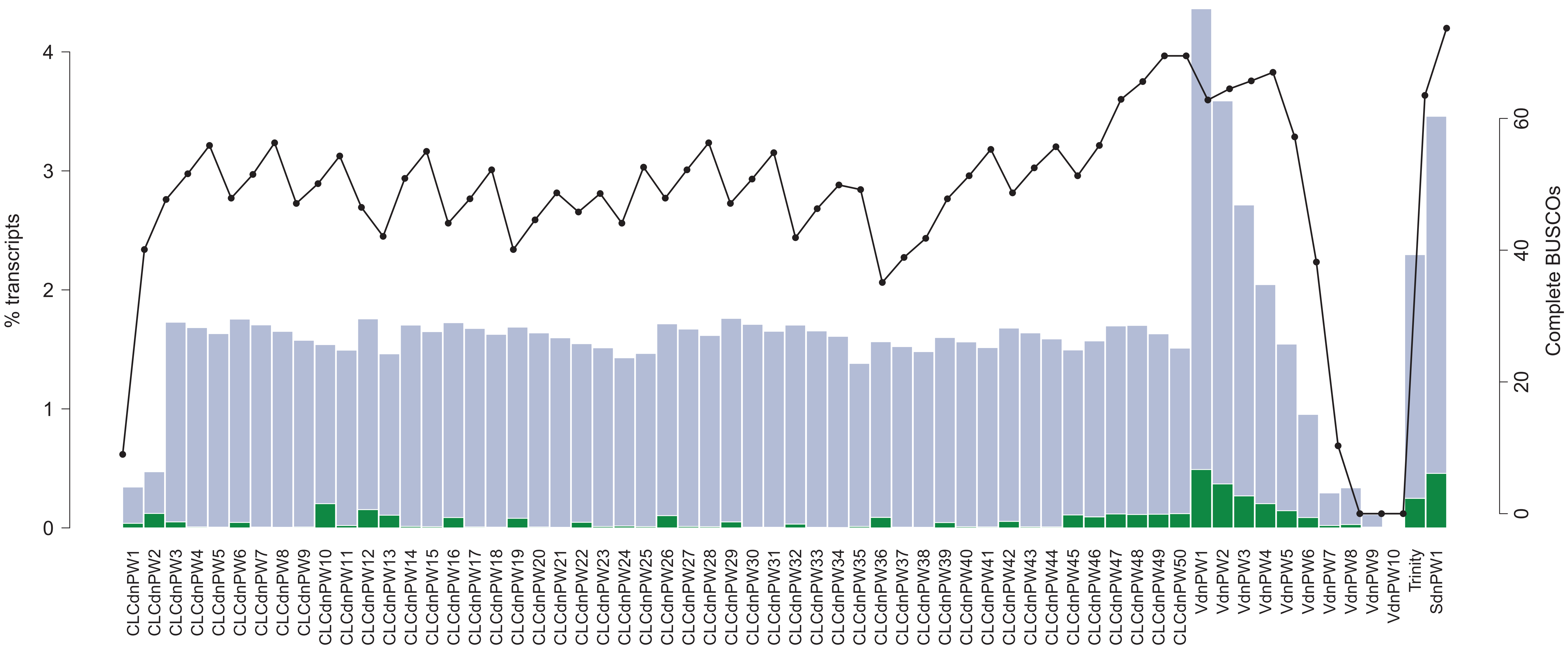

Supplementary Figure 3.

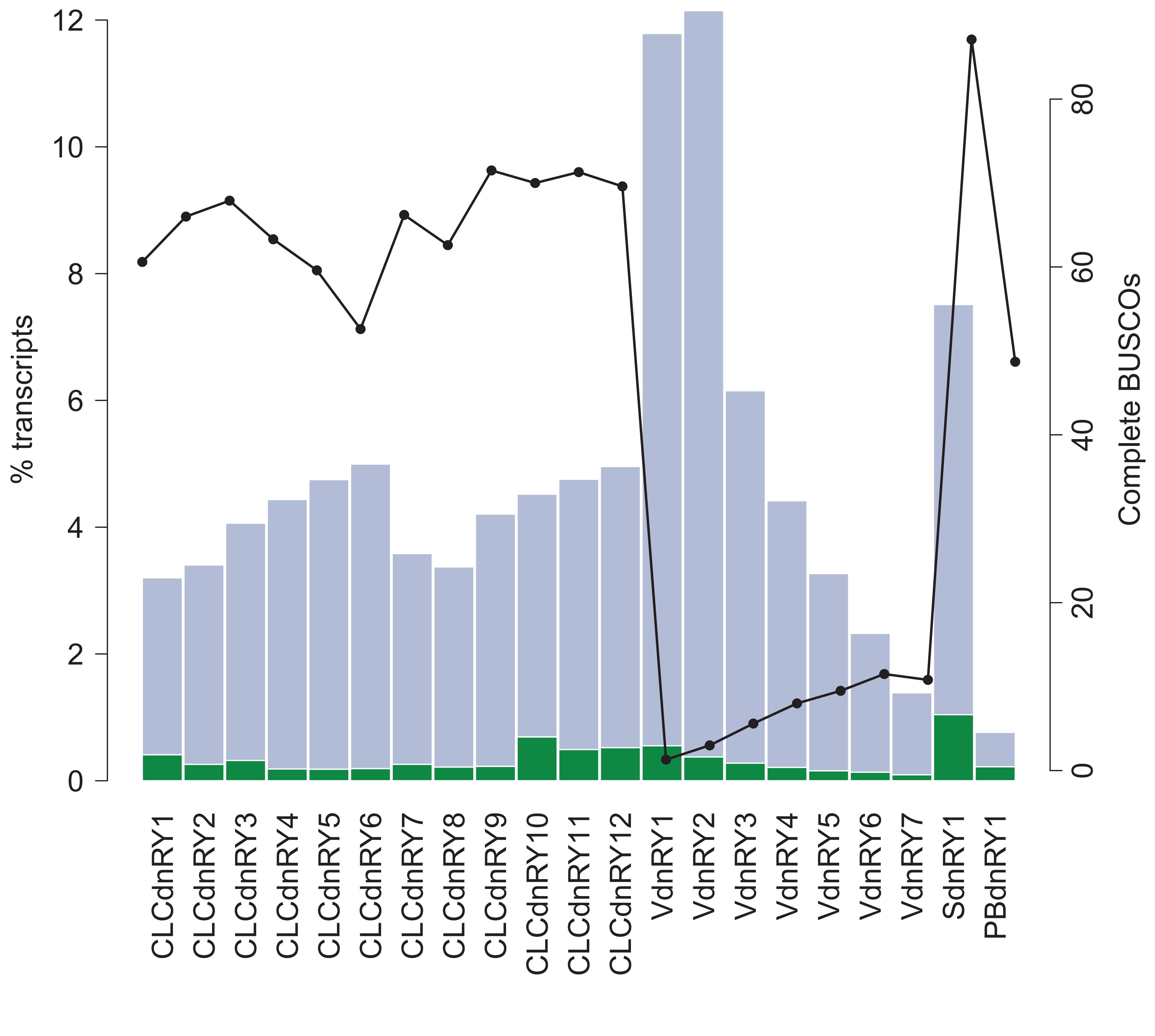

Supplementary Figure 4.

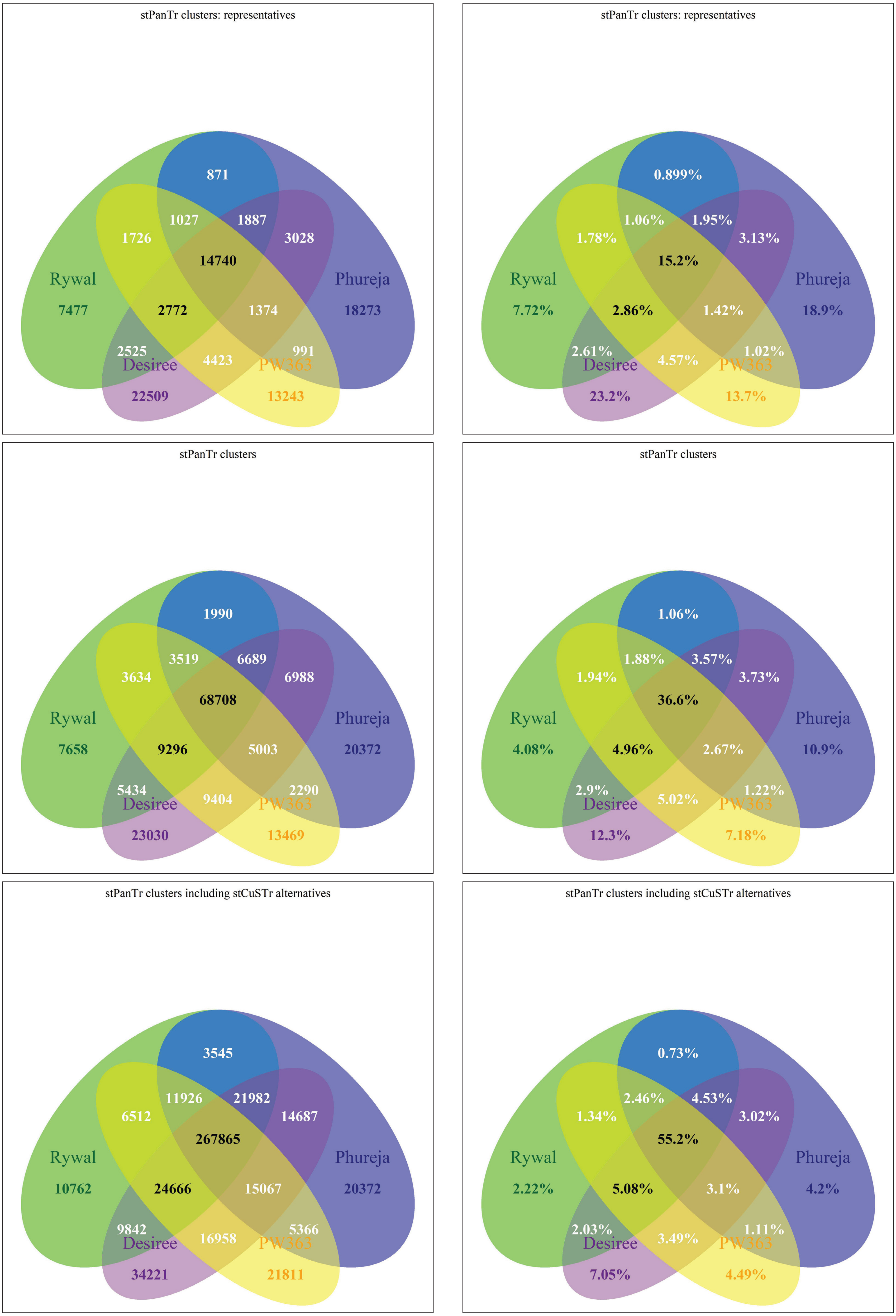

Supplement: Supplementary file 1 — Supplementary Information [file 41597_2020_581_MOESM1_ESM.pdf]
